# Evaluation of quantitative CMR perfusion imaging by comparison with simultaneous ^15^O-water-PET

**DOI:** 10.1007/s12350-019-01810-z

**Published:** 2019-07-16

**Authors:** Tanja Kero, Edvin Johansson, Mathias Engström, Kai M. Eggers, Lars Johansson, Håkan Ahlström, Mark Lubberink

**Affiliations:** 1 Medical Imaging Centre, Uppsala University Hospital, 75185 Uppsala, Sweden; 2 Department of Cardiology, Uppsala University Hospital, Uppsala, Sweden; 3 Medical Physics, Uppsala University Hospital, Uppsala, Sweden; 4 Department of Surgical Sciences/Radiology, Uppsala University, Uppsala, Sweden; 5 Department of Medical Sciences, Uppsala University, Uppsala, Sweden; 6 Antaros Medical, BioVenture Hub, Mölndal, Sweden; 7 GE Healthcare, Waukesha, WI USA

**Keywords:** Myocardial perfusion, myocardial blood flow, cardiac magnetic resonance imaging, positron emission tomography, quantitative modelling

## Abstract

**Background:**

We assessed the quantitative accuracy of cardiac perfusion measurements using dynamic contrast-enhanced MRI with simultaneous ^15^O-water PET as reference with a fully integrated PET-MR scanner.

**Methods:**

15 patients underwent simultaneous DCE MRI and ^15^O-water PET scans at rest and adenosine-stress on an integrated PET-MR scanner. Correlation and agreement between MRI- and PET-based global and regional MBF values were assessed using correlation and Bland–Altman analysis.

**Results:**

Three subjects were excluded due to technical problems. Global mean (± SD) MBF values at rest and stress were 0.97 ± 0.27 and 3.19 ± 0.70 mL/g/min for MRI and 1.02 ± 0.28 and 3.13 ± 1.16 mL/g/min for PET (*P* = 0.66 and *P* = 0.81). The correlations between global and regional MRI- and PET-based MBF values were strong (*r* = 0.86 and *r* = 0.75). The biases were negligible for both global and regional MBF comparisons (0.01 and 0.00 mL/min/g for both), but the limits of agreement were wide for both global and regional MBF, with larger variability for high MBF-values.

**Conclusion:**

The correlation between simultaneous MBF measurements with DCE MRI and ^15^O-water PET measured in an integrated PET-MRI was strong but the agreement was only moderate indicating that MRI-based quantitative MBF measurements is not ready for clinical introduction.

**Electronic supplementary material:**

The online version of this article (10.1007/s12350-019-01810-z) contains supplementary material, which is available to authorized users.

## Introduction

Non-invasive assessment of myocardial perfusion is an important part of the diagnostic strategy and management of patients with known or suspected coronary artery disease (CAD). Clinical perfusion imaging relies mostly on qualitative visual or semi-quantitative assessment of relative myocardial perfusion that can underestimate myocardial blood flow (MBF) in patients with global reduction. Quantification of perfusion overcomes this limitation of qualitative methods and provides incremental value in patients with multi-vessel disease^1^–^4^ and in assessment of microvascular dysfunction.^5^–^7^

^15^O-water PET is considered to be the gold standard for non-invasive quantitative measurements of MBF.^8^–^10^ However, PET is associated with radiation exposure, albeit very limited in the case of short-lived ^15^O, and is not as widely available as MRI. Furthermore, ^15^O-water is freely diffusible and is not, like other PET or SPECT perfusion tracers, trapped in the myocardium. Although assessment of left ventricular volumes and function with ^15^O-water has been demonstrated,^11^,^12^ it is technically challenging.

On the other hand, cardiac magnetic resonance (CMR) is more widely available and is an excellent tool for assessment of cardiac morphology, ventricular volumes and function and myocardial viability. CMR is also increasingly used for myocardial perfusion imaging and has shown excellent diagnostic performance in the detection of obstructive CAD.^13^–^16^ Improvements in acquisition and post-processing methods have now paved way for CMR quantification of MBF. If myocardial perfusion can accurately be quantified with MRI, using a clinically feasible MRI method, this would greatly enhance the availability of quantitative MBF imaging in hospitals where PET with short-lived tracers is unavailable.

Although quantitative perfusion imaging with CMR has been validated against microspheres in animals,^17^–^19^ earlier comparisons of myocardial perfusion measurements with sequential MRI and PET in humans have shown varying results^20^–^24^ which could be due to either physiological or methodological differences. In the last few years, integrated PET-MRI systems have become available, which allow for MBF measurements with CMR and PET simultaneously during the same physiological condition. A recent study compared quantitative myocardial perfusion using simultaneous ^13^NH_3_-ammonia PET and dynamic contrast-enhanced MRI in patients^25^ but to our knowledge, there are no previously published studies on simultaneous ^15^O-water PET and MRI myocardial perfusion quantification in humans.

The aim of the present work was therefore to assess the quantitative accuracy of cardiac perfusion measurements using dynamic contrast-enhanced MRI with simultaneous ^15^O-water PET as reference at rest and during adenosine-induced hyperemia with a fully integrated PET-MR scanner in patients with known or suspected CAD.

## Materials and Methods

### Subjects

15 patients were included in this prospective study. The patients had known or suspected CAD with intermediate pre-test probability of obstructive coronary disease according to ESC Guidelines,^26^ and were referred for a ^15^O-water PET study for evaluation of MBF. Patients were excluded if they had any contraindications to MRI or adenosine, estimated glomerular filtration rate < 60 mL/min or known previous ST-segment elevation myocardial infarction. Written, informed consent was obtained from all subjects and the study was performed with permission from the local Radiation Ethics Committee and the Regional Board of Medical Ethics in Uppsala and in accordance with the Declaration of Helsinki

### Scan Procedure

PET-MR scans were acquired on a 3.0 T PET-MR scanner (Signa PET/MR, GE Healthcare, Waukesha). A 16-channel anterior coil was used in combination with a 14-channel posterior during the MR-scans. System sensitivity is 23 cps/kBq, and the scanner is capable of time-of-flight-PET with a time resolution of circa 370 ps (manufacturer’s specifications and authors’ NEMA measurements).

All subjects underwent simultaneous ^15^O-water PET and gadoterate meglumine (Gd-DOTA) perfusion MRI scans at rest and during adenosine-induced hyperaemia. All the subjects were instructed to abstain from caffeine for 24 hours before imaging.

A 6-min dynamic PET perfusion scan during rest was started simultaneously with the administration of a 10-mL bolus of 400 MBq of ^15^O-water at a flow rate of 0.8 mL/s followed by a bolus of saline (30 mL at 2 mL/s). MRI perfusion imaging was performed during the PET scan; a single bolus of 0.05 mmol/kg body weight Gd-DOTA contrast agent (Dotarem, Guerbet, Roissy Charles de Gaulle Cedex, France) was injected 3 minutes after the start of the PET scan at a flow rate 5 mL/s by a power injector followed by a bolus of saline (25 mL NaCl at 5 mL/s). An ultrafast gradient echo sequence (FGRE Time Course) was used for MRI perfusion imaging using electrocardiography (ECG)-triggered and breath holding technique with the following imaging parameters: TR 3.4 ms, TE 1.4 msec, flip angle 20°, 380 × 304 mm FOV, slice thickness 8 mm, 128 × 102 matrix, pre-pulse delay 120 msec. The perfusion images were acquired for 65 consecutive heartbeats and consisted of 3 short-axis slices (basal, midventricular, and apical).

Functional MRI-imaging was obtained between the rest and stress PET-scans with a balanced SSFP cine sequence covering the left ventricular myocardium from apex to base in 8-mm-thick short-axis slices with 2.0 mm gap. After the functional MRI-images, a dynamic PET scan and an MRI perfusion scan (as described previously during resting state) were performed during adenosine-induced hyperaemia. Adenosine infusion 140 μg × kg^−1 ^× min^−1^ was started 2 min prior to the stress scan and continued during the PET and MRI scan time.

To correct for photon attenuation, a two-point Dixon sequence was acquired in breath hold during the resting PET scan and during the hyperaemic PET scan. This sequence enabled segmentation of fat and water tissue, lungs and air, which form the basis for creation of the MRI-based attenuation map. The arms, which are not covered in the MRI images, are added to the attenuation map from non-attenuation corrected TOF-PET data.^27^ PET- images were reconstructed using OSEM into 128 × 128 pixel images and a FOV of 53.4 cm, using the cardiac protocol recommended by the scanner manufacturer.

### Data Analysis

The MRI and the PET images were analysed and segments were defined separately. The right ventricular insertion was used as reference point. Myocardial segment VOIs were drawn over the left ventricle based on the 17-segment model of the American Heart Association^28^ and segmental signal and activity vs. time curves were extracted. Myocardial blood flow was calculated for the entire left ventricle and for three regions corresponding to the coronary artery territories.

Regions of interest were defined in the MRI images using the software package Segment (Medviso, Lund, Sweden).^29^ The left ventricular endo- and epicardial borders were manually delineated for both rest and stress data, and signal vs. time curves were then generated for myocardial tissue and for ventricular blood. The PET data was analysed semi-automatically using Cardiac VUer software,^30^ generating MBF-values for the entire left ventricle and in three regions corresponding to the coronary artery territories.

MRI signal was converted to Gd concentrations and MBF was calculated according to the methods described in detail in "Appendix".

### Statistical Analyses

Statistical analyses were performed using the IBM SPSS Statistics (version 25.0 for Macintosh, SPSS, Chicago, Illinois) and GraphPad Prism (version 6 for Windows, GraphPad Software, La Jolla California).

Continuous variables are presented as mean values ± standard deviation (SD), except were stated. Comparison of the global MBF and MFR values was performed by paired T-test. Correlations and agreement between MRI- and PET-based MBF and MFR values were assessed using Spearman's rank correlation coefficient and Bland-Altman analysis. A two-sided *P* value of less than 0.05 was considered significant.

## Results

### Study Population

15 patients were included in the study. Table 1 shows baseline characteristics. The patients had known (*n* = 7) or suspected CAD (*n* = 8; 20% to 68% clinical pre-test probability according to ESC Guidelines).^26^ Three subjects were excluded due to technical problems during the PET-MR scan: one subject due to contrast injector failure and two subjects due to ECG-gating failure during the MRI-perfusion scan. The scan protocol was performed within approximately one hour. Time between rest and stress perfusion scans was 33 min (31 to 36 min). CMR showed normal global and regional systolic function in all 12 subjects, with a mean ejection fraction (EF) of 66% ± 6%. The heart rate and the systolic blood pressure increased from rest to stress: from 66 ± 9 bpm to 82 ± 13 bpm and from 131 ± 15 mmHg to 138 ± 18 mmHg at stress (*P* < 0.0005 and *P* = 0.01, respectively).

**Table 1:** Patient characteristics

Age (years)	66 (range 51–75)
Male	9 (60%)
Current smoking	0 (0%)
Previous smoking	3 (20%)
Previous CABG	1 (7%)
Previous PCI	6 (40%)
Diabetes	3 (20%)
Hypertension	9 (60%)
Hyperlipidemia	10 (67%)
Betablockers	4 (27%)
Statins	11 (73%)
ACE-inhibitors/ ARB	10 (67%)
Calcium channel blockers	3 (20%)
Nitrate	3 (20%)
Anticoagulants	11 (73%)

*CABG*, coronary artery bypass grafting; *PCI*, percutaneous coronary intervention, *ACE*, angiotensin converting enzyme; *ARB*, angiotensin receptor blocker

### Myocardial Perfusion

**Figure 1:**
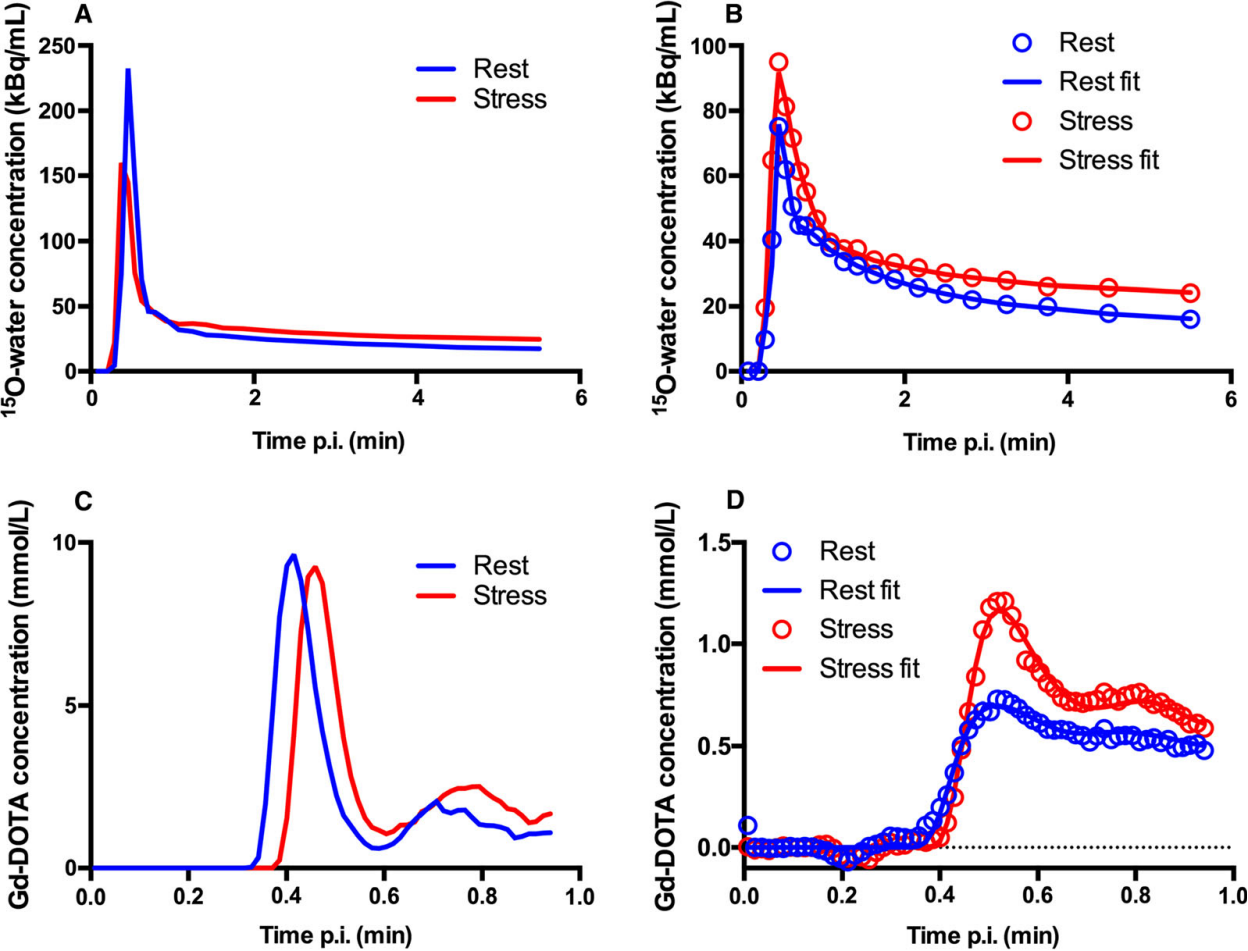
PET (**A**, **B**) and MR (**C**, **D**) arterial input curves (**A**, **C**) and whole myocardium time-activity curves and model (**B**, **D**) in a typical patient

An example of a myocardial time activity curve (TAC) from a typical patient together with corresponding fits and input curves from MRI and PET perfusion analysis is shown in Figure 1. Segmental MBF and MFR values were excluded in 6 regional segments due to unreliable fits of the 1TCM+PS model during analysis of MRI perfusion data.

**Figure 2:**
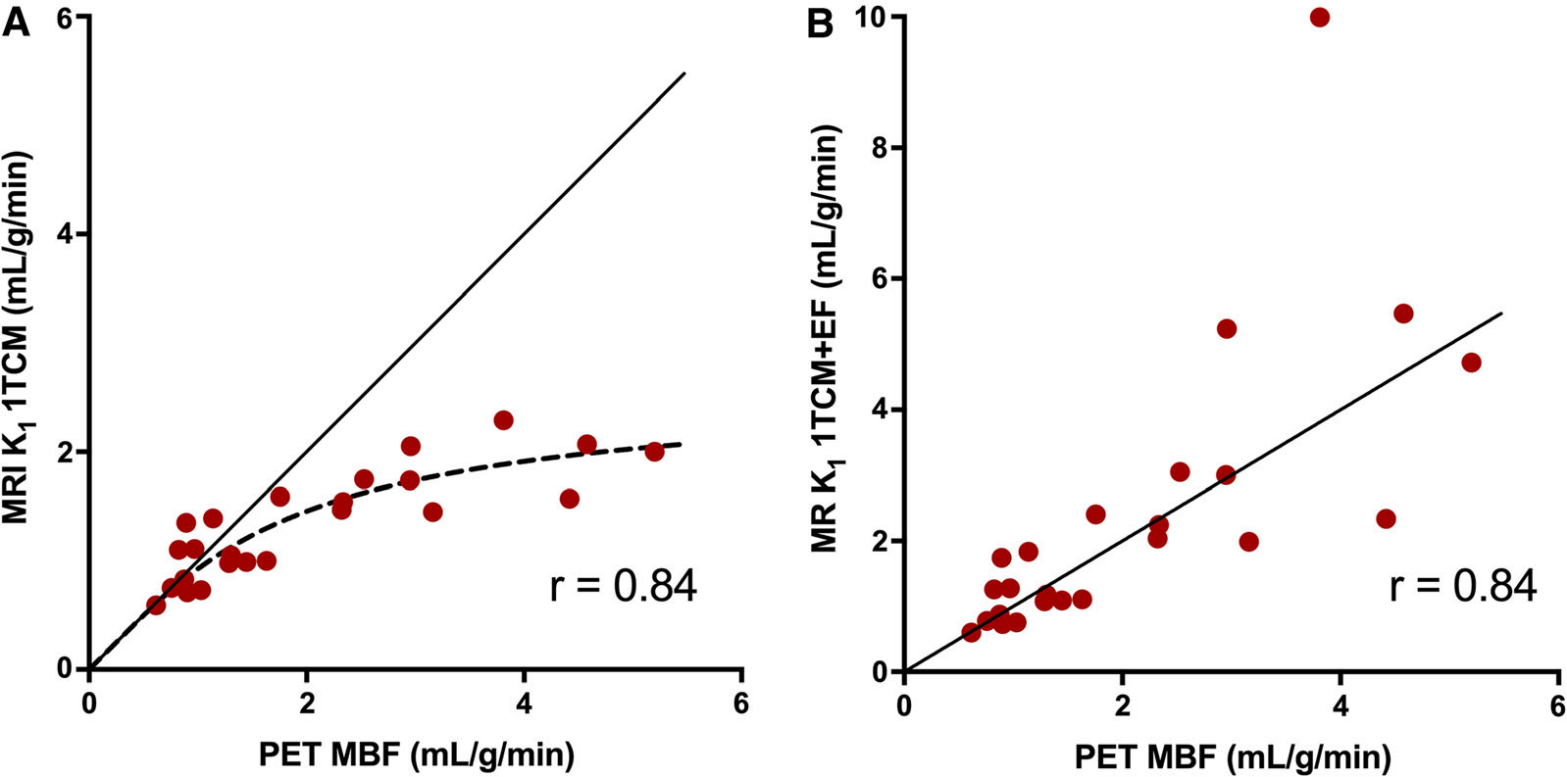
Correlation of MR 1TCM-based global *K*_1_ vs PET based MBF (**A**). Correlation of MR 1TCM-based global *K*_1_ with correction for extraction fraction (EF) vs PET-based MBF (**B**)

Using a 1TCM for MRI and PET perfusion analysis, the relationship between MRI-based perfusion related parameter, *K*_1_, and PET-based MBF, is shown in Figure 2a; the MR-based *K*_1_ values underestimated perfusion above approximately MBF > 1 mL/min/g, as compared to PET MBF. Figure 2b shows the relationship between MRI-based *K*_1_ and PET-based MBF when an extraction fraction correction has been applied to the MRI-based *K*_1_-values. The permeability surface area product of Gd-DOTA was estimated to be 2.6 mL/g/min. Although perfusion values > 1 mL/min/g were no longer systematically underestimated after this correction, the extraction fraction-corrected *K*_1_ values correlated poorly with PET MBF at high values.

**Figure 3:**
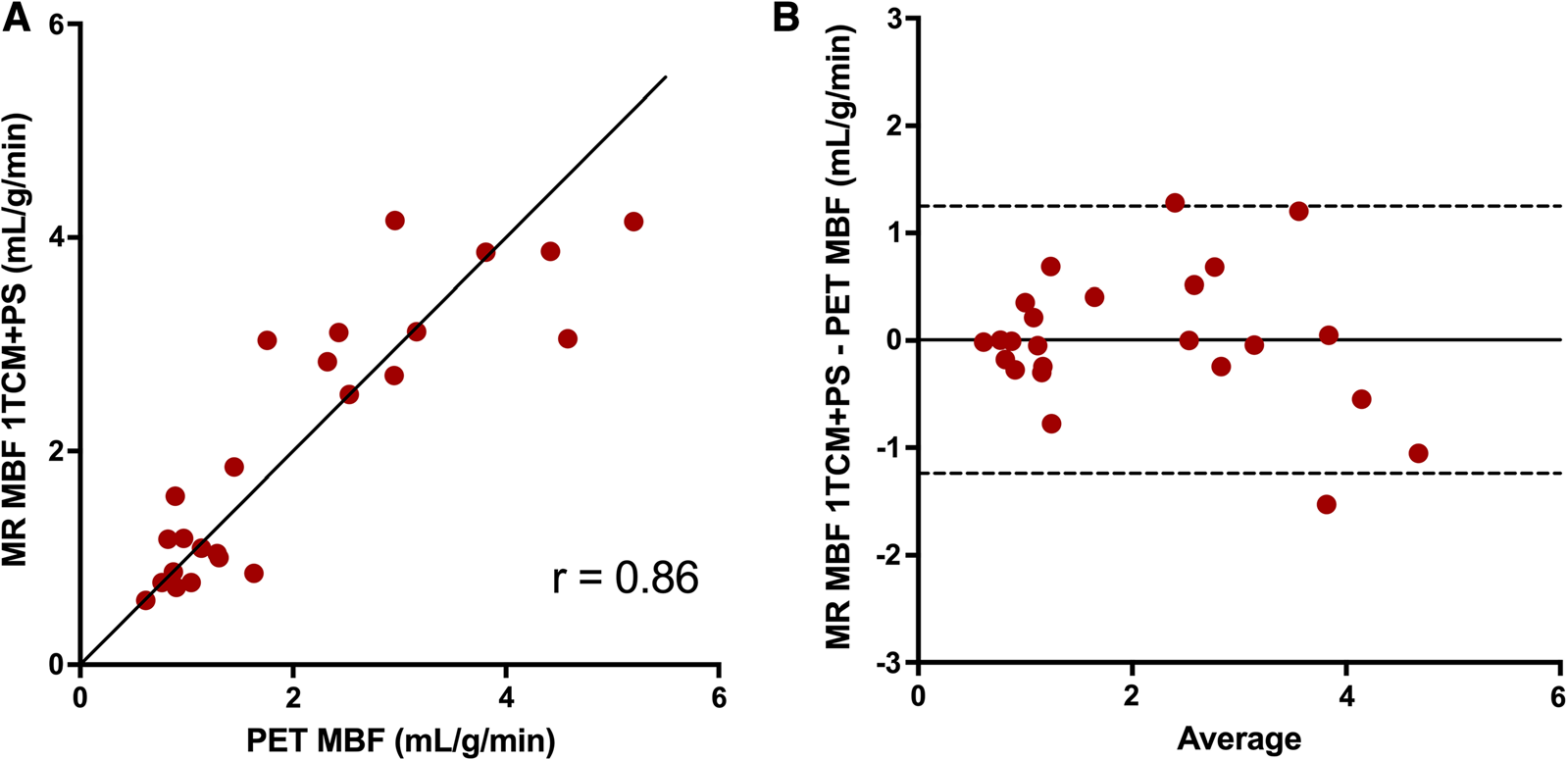
Correlation (**A**) and Bland–Altman plots (**B**) of MR-based global MBF vs PET-based global MBF at rest and stress. The solid line in **A** is line of identity. The solid lines in **B** indicate the mean difference (bias), whereas the dashed lines show the limits of agreement). Bias (limits of agreement) in b is 0.01 (− 1.24 to 1.25)

**Figure 4:**
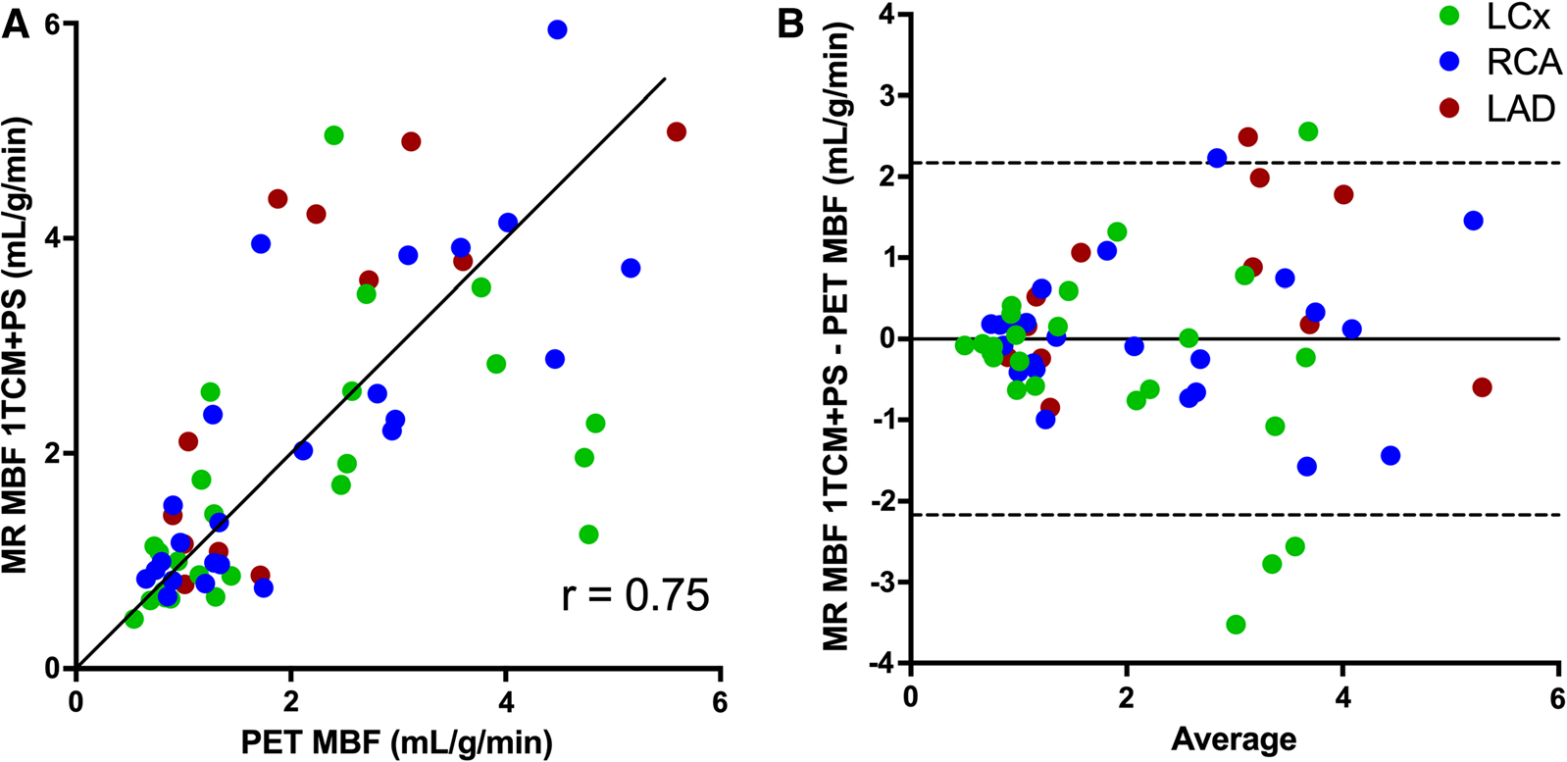
Correlation (**A**) and Bland–Altman plots (**B**) of MR-based regional MBF vs PET-based regional MBF at rest and stress. The solid line in **A** is line of identity. The solid line in **B** indicate the mean difference (bias), whereas the dashed lines show the limits of agreement. Bias (limits of agreement) in b are 0.00 (− 2.17 to 2.17). Correlation for the three separate coronary artery regions are: LAD *r*  =  0.78 (red), RCA r  =  0.82 (blue), LCx *r*  =  0.53 (green)

**Figure 5:**
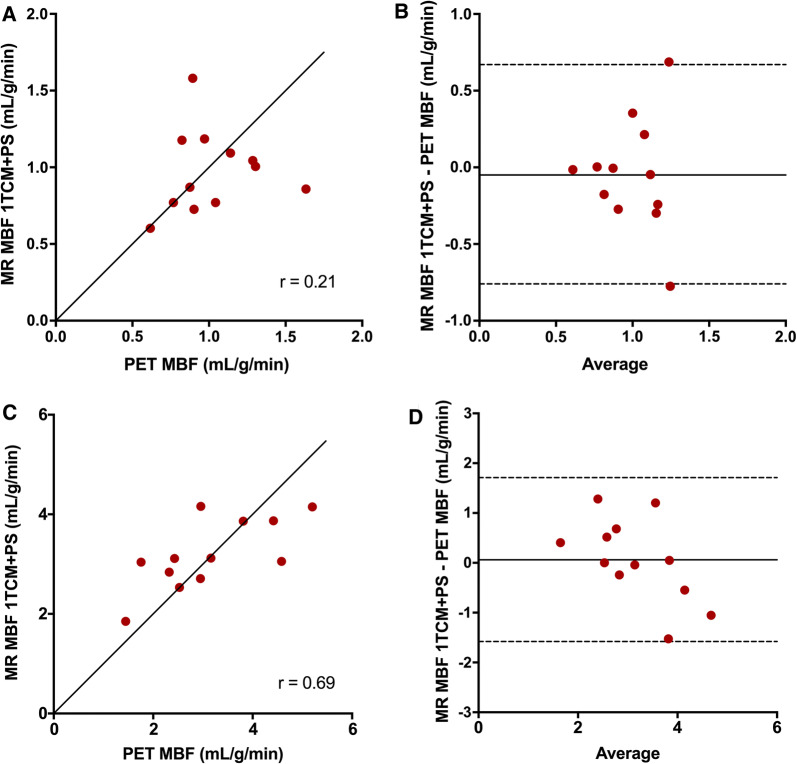
Correlation and Bland–Altman plots of MR-based global MBF vs PET-based global MBF at rest (**A**, **B**) and at stress (**C**, **D**). The solid lines in **A** and **C** are lines of identity. The solid lines in **B** and **D** indicate the mean differences (bias), whereas the dashed lines show the limits of agreement. Bias (limits of agreement) in **B** are − 0.05 (− 0.76 to 0.67) and in **D** 0.06 (− 1.58 to 1.71)

When using an MRI-analysis model that included the permeability surface area product (PS) in order to correct for the low extraction of Gd-DOTA, global mean (± SD) MBF values at rest and stress were 0.97 ± 0.27 and 3.19 ± 0.70 mL/g/min for MRI and 1.02 ± 0.28 and 3.13 ± 1.16 mL/g/min for PET (*P* = 0.66 and *P* = 0.81). Mean PS was 2.91 ± 0.37 mL/g/min. The relationships between MRI-based and PET-based global and regional MBF values are shown in Figures 3 and 4. The correlations between global and regional MRI- and PET-based MBF values were strong (*r* = 0.86 and *r* = 0.75, *P* < 0.0005 for both). Although the biases were negligible for both global and regional MBF comparisons (0.01 and 0.00 mL/min/g, respectively), the limits of agreement were wide for both global and regional MBF (− 1.24 to 1.25 and − 2.17 to 2.17), with larger variability for higher MBF-values. The relationships between MR-based and PET-based global MBF values at rest and at stress compared separately are shown in Figure 5. The MBF values at rest did not correlate between MRI and PET (*r* = 0.21, *P* = 0.51) while the correlation was moderate for stress MBF values (*r* = 0.69, *P* = 0.013). Biases were negligible for both rest and stress MBF comparisons (0.06 and − 0.05) but the limits of agreement were wide for stress MBF values (− 1.58 to 1.71).

Global mean (± SD) MFR values were 3.44 ± 0.97 for MRI and 3.05 ± 0.76 for PET (*P* = 0.83).

**Figure 6:**
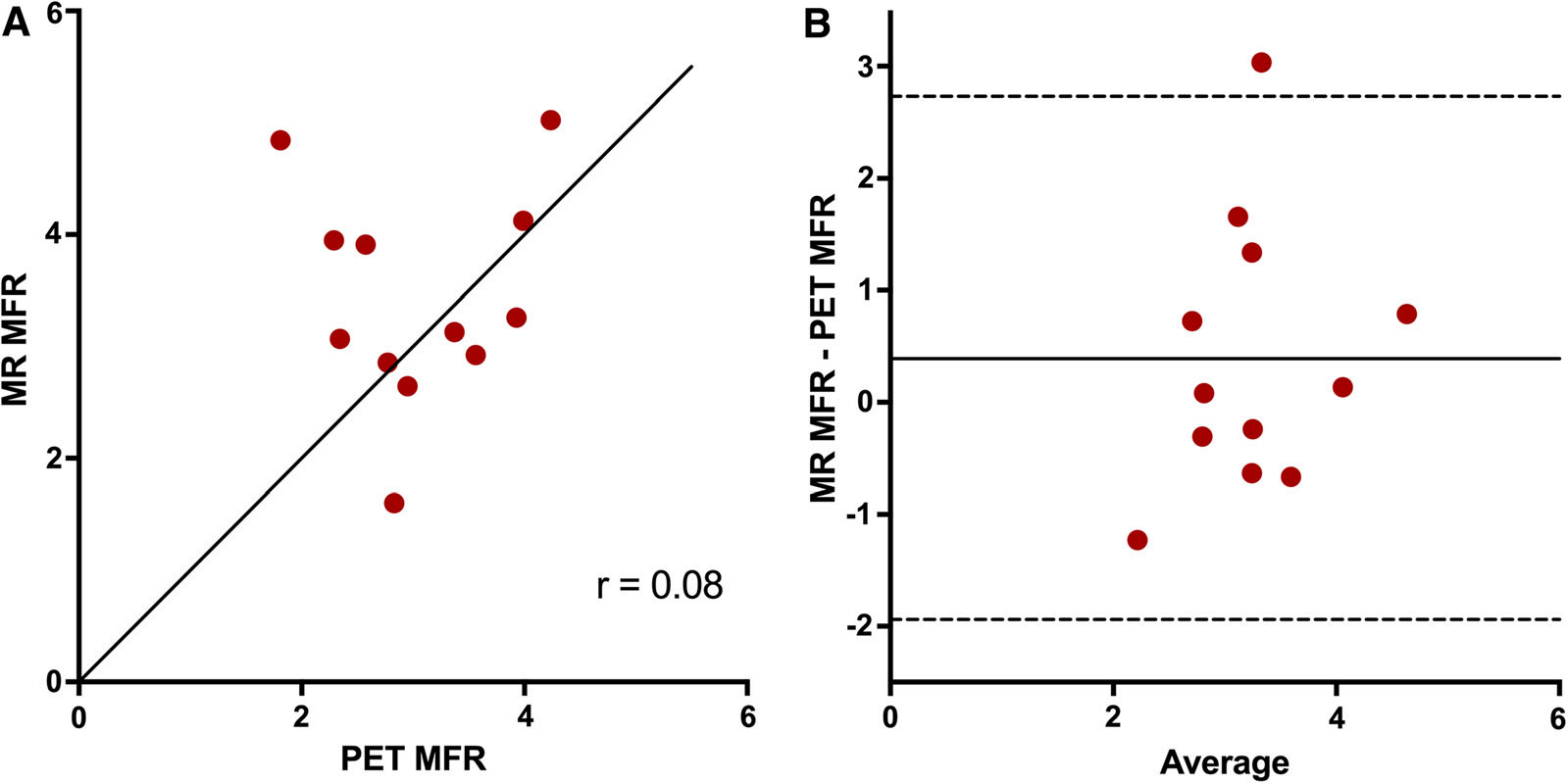
Correlation (**A**) and agreement (**B**) of MR-based global MFR vs PET-based global MFR. The solid line in a is line of identity. The solid line in** B** indicates the mean difference (bias), whereas the dashed lines show the limits of agreement. Bias (limits of agreement) in b are 0.39 (− 1.94 to 2.73)

The relationships between MRI-based and PET-based global and regional MFR are shown in Figure 6. There was no significant correlation between MRI- and PET-based MFR (*r* =  0.08, *P* = 0.80). The bias for MFR was 0.39 and the limits of agreement were wide (− 1.94 to 2.73).

## Discussion

The present study assessed the quantitative accuracy of cardiac perfusion measurements using dynamic contrast-enhanced MRI with simultaneous ^15^O-water PET as reference at rest and during adenosine-induced hyperemia with a fully integrated PET-MR scanner. Although a good correlation and a negligible bias between MRI-based and PET-based MBF was found, the agreement was only moderate, with a large variability, especially for the higher MBF values.

^15^O-water PET, used in the current study, is considered to be the gold standard for non-invasive quantitative measurements of myocardial blood flow (MBF).^8^–^10^^15^O-water is metabolically inert and freely diffusible allowing for accurate quantification also at high flow rates, thus being the optimal reference PET-tracer method in comparison studies. Both the PET tracers ^13^NH_3_-ammonia and ^82^Rb are currently accepted for clinical myocardial perfusion imaging. With appropriate kinetic models and corrections for metabolites and extraction fraction ^13^NH_3_-ammonia can accurately quantify MBF.^9^,^31^^82^Rb, just like gadolinium, has a low and non-linearly related extraction fraction from blood to myocardial tissue, and quantification of MBF highly depends on accurate modelling and correction of the extraction fraction. However, although comparisons of MBF quantified with ^82^Rb and ^15^O-water^32^,^33^ and with ^82^Rb and ^13^NH_3_-ammonia^34^ have shown promising results with good correlations, the limits of agreement seems wider for high flow values indicating that the correction algorithms also introduce error. Most of the previously published studies comparing CMR and PET myocardial perfusion in humans were performed at different time points and in separate MRI and PET scanners. Furthermore, most studies have been either semiquantitative^35^,^36^ or have included healthy volunteers only.^20^,^22^,^37^ Quantitative studies including patients with CAD have all used different MRI methods and PET tracers,^21^,^23^,^24^,^38^ making direct comparisons difficult.

Table 2 summarizes CMR and PET studies comparing quantitative myocardial perfusion.

**Table 2:** Summary of studies comparing quantitative myocardial perfusion with MRI and PET

Study	Study population	Sequential/simultaneous PET and MRI scans	PET tracer	MRI perfusion acquisition approach	MRI perfusion analysis model
Pärkkä et al.^22^	18 healthy males	Sequential Separate days	^15^O-water	Single bolus, single sequence	1TCM
Fritz-Hansen et al.^20^	10 healthy males	Sequential 3 - 12 days between MRI and PET	^13^N-ammonia	Single bolus, single sequence	1TCM
Pack et al.^37^	4 healthy volunteers, one heart-transplant patient	Sequential 1–6 months between MRI and PET	^13^N-ammonia	Single bolus, single sequence	Independent deconvolution
Morton et al.^21^	38 patients with known or suspected CAD	Sequential 3 ± 6 days between PET and MRI	^13^N-ammonia	Dual bolus	Fermi deconvolution
Qayyum et al.^23^	14 patients with CAD	Sequential 6.6 ± 30.3 days	^82^Rb	Single bolus, single sequence	Tikhonov´s deconvolution
Tomiyama et al.^38^	10 CAD patients	Sequential 11.9 ± 8.78 days	^15^O-water	Single bolus, single sequence	1TCM
Engblom et al.^24^	21 patients with CAD	Sequential 4–5 h between MRI and PET	^13^N-ammonia	Single bolus, dual sequence	Distributed model
Kunze et al.^25^	29 patients with known or suspected CAD	Simultaneous	^13^N-ammonia	Single bolus, dual sequence	Four different deconvolution methods
Kero 2019 (current work)	12 patients with known or suspected CAD	Simultaneous	^15^O-water	Single bolus, single sequence	1TCM, direct estimation of PS and MBF

Study	MBF rest MRI and PET (mL/g/min)	MBF stress MRI and PET (mL/g/min)	Comparison of MRI-based and PET-based MBF (correlation and Bland-Altman comparison if available)	MFR MRI and PET	Comparison of MRI-based and PET-based MFR (correlation)
Pärkkä et al.^22^	0.71 ± 0.24 0.92 ± 0.26	1.72 ± 0.67 (*K*_1_) 3.76 ± 1.21	Regional MBF *r* = 0.80	2.51 ± 0.95 4.32 ± 1.78	Regional MFR *r* = 0.46
Fritz-Hansen et al.^20^	0.80 ± 0.20 0.71 ± 0.16	1.83 ± 0.56 (*K*_1_) 2.03 ± 0.67	Regional MBF difference (stress-rest) *r* = 0.86 Regional MBF bias -0.28, LoA -0.45 – (-0.12)	2.4 ± 0.8 2.9 ± 0.8	Global MFR *r* = 0.7
Pack et al.^37^	1.03 ± 0.76 0.80 ± 0.24	2.97 ± 1.59 3.04 ± 1.14	Regional MBF *r* = 0.85 Regional MBF bias 0.12	3.2 ± 1.7 3.7 ± 0.7	n/a
Morton et al.^21^	MBF values reported separately for different subject groups and coronary territories		Regional MBF rest *r* = 0.32 Regional MBF stress *r* = 0.37	MFR values reported separately for different subject groups and coronary territories	Regional MFR *r* = 0.75
Qayyum et al.^23^	1.7 ± 0.49 0.75 ± 0.37	2.65 ± 1.77 1.90 ± 1.61	Global MBF difference (stress-rest) *r* = 0.81 Global MBF bias -0.11 ± 0.98	n/a	Global MFR *r* = 0.89 Regional MFR *r* = 0.82
Tomiyama et al.^38^	0.76 ± 0.10 0.71 ± 0.11	3.04 ± 0.82 3.09 ± 0.97	Global MBF *r* = 0.96 Regional MBF *r* = 0.92	4.13 ± 1.33 4.46 ± 1.43	Global MFR *r* = 0.93 Regional MFR *r* = 0.83
Engblom et al.^24^	MRI rest and stress MBF range 0.6-3.8 PET rest and stress MBF range 0.6-4.0		Global MBF *r* = 0.92, rest bias 0 ± 0.2, stress bias -0.1 ± 0.5 Regional MBF *r* = 0.83, bias -0.1 ± 0.6	n/a	Global MFR *r* = 0.69 Regional MFR *r* = 0.57
Kunze et al.^25^	0.94 ± 0.23 0.78 ± 0.23	1.98 ± -0.49 1.89 ± 0.41	Slice average MBF *r* = 0.91 Regional MBF *r* = 0.84	2.09 2.53	Slice average MFR *r* = 0.60
Kero 2019 (current work)	0.97 ± 0.27 1.02 ± 0.28	3.19 ± 0.70 3.13 ± 1.16	Global MBF *r* = 0.86, rest bias 0.06, stress bias -0.05, stress LoA -1.58 – 1.71 Regional MBF *r* = 0.75	3.44 ± 0.97 3.05 ± 0.76	Global MFR *r* = 0.08, bias 0.39, LoA -1.94 – 2.73

*1TCM*, single-tissue compartment model; *LoA*, limits of agreement

Pärkkä et al.^22^ calculated a perfusion-related parameter, the unidirectional influx constant (*K*_i_), which correlated significantly with ^15^O-water based PET rest and stress perfusion (*r* = 0.80).^22^ However, absolute stress values of MRI-based Ki were lower than PET based MBF, which is in line with our results when analysing our MRI data using the 1TCM, showing an underestimation of perfusion values > 2 mL/min/g, due to the lower extraction fraction of Gd-DOTA as compared to ^15^O-water. Applying a correction for the extraction fraction of Gd-DOTA is technically possible but introduces uncertainty and possible error as the extraction fraction may vary according to the coronary flow.^39^,^40^ Pärkkä et al. considered the extraction of Gd to be constantly 50%, and after correcting for extraction the MRI-based stress MBF values became comparable to PET MBF, whereas the resting MBF values became higher with MRI than with PET. Tomiyama et al.^38^ estimated Renkin-Crone parameters with the relationship between *K*_1_ values from MRI and MBF values from ^15^O-water PET and then calculated a correction for extraction fraction for Gd-DTPA. When applying this correction to the MRI perfusion analysis, they reported very high correlations between MRI-based and PET-based perfusion values (*r* = 0.96 for global rest and stress MBF) without overt under- or overestimation of absolute perfusion values. When attempting a similar method for extraction fraction correction on our data, the MRI stress perfusion values indeed increased, but the correction algorithm caused a larger variation in stress MBF values (Figure 2b), as high correction factors also multiply the noise of the measurements leading to larger scatter of the values. The 1TCM+PS model allows for direct estimation of MBF by included Renkin-Crone parameters for permeability-surface area, thus implicitly correcting for extraction. This resulted in comparable global mean MBF values for MRI and PET at both rest and stress (0.97 ± 0.27 vs 1.02 ± 0.28 mL/g/min, *P* = 0.66) and (3.19 ± 0.70 vs 3.13 ± 1.16 mL/g/min, *P* = 0.81), with a strong correlation for rest and stress values together (*r* = 0.86, *P* < 0.0005 for global MBF values), although with large variability, seen as wide limits of agreement (− 1.24 to 1.25 mL/min/g) in a Bland–Altman comparison, shown in Fig 3b. This cannot be attributed to differences in the physiological state as the PET and MRI perfusion was measured simultaneously, but must then depend on differences between the modalities, tracers and/or analysis methods.

The reproducibility of ^15^O-water PET myocardial perfusion has been assessed and found to be good.^41^ Another comparison between sequential ^15^O-water PET MBF measurements in a PET-CT and in a PET-MR also yielded high correlation and agreement (ICC = 0.98, bias − 0.04, limits of agreement − 0.73 to 0.65 mL/min/g for rest and stress regional MBF values),^42^ although both physiological differences and different scanners might have influenced the measurements. Reproducibility of quantitative cardiac perfusion measurements with MRI has been reported to be good or at least moderate.^43^–^47^ Direct comparison with the reproducibility of PET-based myocardial perfusion measurements is hampered by different measures of repeatability used in different studies and wide time ranges between the repeated measures in some studies. However, the repeatability coefficients reported in some MRI studies^44^,^47^ are somewhat higher and the ICC somewhat lower^44^,^46^ than in the previously mentioned PET-studies.^41^,^42^

Morton et al.^21^ reported weak correlations for absolute perfusion values when comparing rest and stress perfusion values separately (*r* = 0.32 and *r* = 0.37, respectively). In our study, the MBF values at rest did not correlate between MRI and PET (*r* = 0.21, *P* = 0.51) while the correlation was moderate for stress MBF values (*r* = 0.69, *P* = 0.013). Pärkkä and Tomiyama et al. did not analyzs rest and stress perfusion separately; however, a weak (or non-significant) correlation for rest MBF and a moderate correlation for stress MBF values was likely present, as indicated by the scatterplots.^22^,^38^ From a statistical point of view, analysing rest and stress values separately is probably more correct.

MFR is a commonly used measure in the diagnosis of CAD. Morton et al.^21^ and Qayyum et al.^23^ reported a good correlation for MRI and PET MFR values (*r* = 0.75 and *r* = 0.89, respectively), while the correlation for MFR values was moderate in the study by Pärkkä et al. (*r* = 0.46).^22^ Tomiyama et al.^38^ reported a very strong correlation for MFR values (*r* = 0.93), but this finding seems to depend on a few extreme MFR values. Myocardial perfusion at rest is depending on heart rate and systolic blood pressure, and as MFR values depend on both baseline and hyperemic MBF, in sequential comparison studies a larger variation in values is expected for both rest MBF and MFR than for stress MBF values. In our data however, there was no significant correlation between MRI- and PET-based based MFR values (*r* =  0.08, *P* = 0.80), which in this case cannot be explained by physiological differences between MRI and PET-scans, but must be solely technical. Although MFR is a measure commonly used in the diagnosis of CAD, several PET studies have shown that absolute MBF at stress is superior to perfusion reserve in the detection of hemodynamical significant CAD.^48^–^51^

A recent study by Engblom et al.^24^ reported very good correlation and agreement between MBF values from MRI and PET (*r* = 0.92, − 0.1 ± 0.6 mL/min/g for global rest and stress MBF values analysed together). The MRI method used was based on a single-bolus, dual sequence method and MBF was quantified by a recently developed automated perfusion mapping technique based on a distributed blood-tissue exchange model optimized for MRI by the use of several integrated corrections.^52^ Besides estimation of extraction fraction, the MRI technique and analysis model Engblom et al. used were also optimized to achieve linearity between the measured blood signal and contrast agent concentration. The dual sequence approach used by Engblom et al.,^52^ which separately optimizes the imaging for blood and for myocardium addresses several technical challenges and possible causes of error in quantification of myocardial perfusion and further has a good reported repeatability.^53^ A dual sequence protocol is currently not available for our MRI-system, and in our study, we used an MRI imaging protocol for perfusion that was based on a single MRI sequence for blood and for myocardium. Dual sequence protocols are expected to become available in clinical routine for several MRI scanners, and could possibly improve quantitative perfusion imaging by avoiding signal saturation effects.

Recently Kunze et al.^25^ published the first comparison of quantitative myocardial perfusion using simultaneously acquired PET and dynamic contrast-enhanced MRI with a dual sequence approach on an integrated PET-MRI scanner. Although the absolute flow values were strongly correlated (*R*^2^ = 0.82 for slice-average and *R*^2^ = 0.7 for regional MBF values), the cohort average MBF values at rest were higher for MRI than for PET and subsequently the perfusion ratios were also lower for MRI than for PET. The authors used both arterial and tissue hematocrite fractions in the perfusion MR analysis algorithm and suggested that differences in perfusion ratios between PET and MRI could possibly be corrected using different hematocrite corrections for rest and stress separately.

Accurate quantification of MBF with MRI is challenging due to the non-linear relationship between signal intensity and gadolinium contrast agent concentration.^54^,^55^ In order to avoid signal saturation effects low contrast doses can be used. In our study, we used 0.05 mmol/kg Gd-DOTA and did not find any evidence of flattening of the bolus peak by saturation effects. The same Gd-dose has been used by others,^22^ who found that with Gd-bolus doses up to this level, the increase in the peak concentration was proportional to the given dose, suggesting insignificant saturation of signal. If saturation effects exist and are neglected the myocardial perfusion is overestimated, which is also not apparent in our results. A dual bolus technique^56^ has also been proposed to avoid saturation of the MRI signal during imaging of the Gd-DOTA contrast bolus for the input function, but this prolongs imaging and the possibility of changes between the two bolus acquisitions, which might affect the results. Other possible sources of bias and errors are patient motion, B1-field variation in combination with saturation fluctuations during the bolus passage and saturation recovery variations due to varying cardiac cycle lengths. *T*2* decay is another possible source of MRI signal loss that might affect the results but is not expected to be strong in the current experiment set-up.

Our study, as well as a number of other recent studies, shows a good correlation, with negligible systematic bias, between ^15^O-water PET and CMR perfusion values. However, the limits of agreement between PET- and CMR-based MBF values in Bland-Altman analysis are much wider than those found for test-retest studies with ^15^O-water. For example, in a recent study with two rest-test protocols with ^15^O-water on two different scanners, limits of agreement for combined rest and stress regional MBF values were circa ± 0.7 mL/g/min for regional values, compared to 2.2 mL/g/min in the present work and ± 1.1 mL/g/min in the work by Engblom et al. which was done using an MRI-method which is not available on our scanner. In addition to this, whilst PET MBF analysis can be done very robustly and nearly automatically within minutes using currently widely available software packages, MRI analysis appears to be much more time-consuming, operator dependent, and error-prone.

### Study Limitations

The major limitation of this current study is that the sample size is small and the results should be confirmed in a further study involving more patients.

Another limitation is that this study did not include ICA or CCTA and thus no comparisons between myocardial perfusion measurements and coronary angiography were performed. However, several recent studies have described the diagnostic accuracy of ^15^O-water PET in comparison with coronary angiography.^49^,^57^–^59^

No late gadolinium enhancement (LGE) imaging was performed to assess the presence of myocardial scars, which might have influenced myocardial perfusion; however, the patients did not have any known myocardial infarctions and the left ventricular systolic function was normal in all subjects, why at least large infarctions were less likely. Different acquisition and post-processing methods inevitably result in differences in the myocardial segmentation between modalities. With PET the whole left ventricle was covered while MRI captured three 8-mm-thick short-axis slices in the left ventricle with gaps between the slices.

## New Knowledge Gained

The correlation between simultaneous quantitative MBF measurements with single bolus DCE MRI and ^15^O-water PET measured in an integrated PET-MRI is good but the agreement is only moderate. The variation between the MBF values is due to technical differences between the modalities, tracers, and/or analysis methods.

## Conclusion

Quantification of myocardial perfusion with MRI is technically challenging and depends on several correction algorithms that can lead to large variability of the MBF values. Although MRI analysis likely can be automated in similar ways as PET analysis, the relatively poor agreement with ^15^O-water PET shows that MRI-based quantitative MBF measurements based on widely available acquisition protocols are not ready for clinical introduction.

### Electronic supplementary material

Below is the link to the electronic supplementary material.

Supplementary material 1 (PPTX 4481 kb)

## Appendix

### Calculation of Gd Concentrations

To convert the MRI signal to Gd concentrations, the following steps were taken. The signal intensity for a spoiled gradient echo sequence can be described by:

$$ S = k\frac{{\left( {1 - e^{{ - T_{\text{R}} /T_{ 1} }} } \right){ \sin }\alpha }}{{1 - \left( {{ \cos }\alpha } \right)e^{{ - T_{\text{R}} /T_{ 1} }} }}e^{{ - T_{\text{E}} /T_{2}^{*} }} $$

Here, *T*_R_ is the repetition time (3.4 ms), $\alpha$ is the flip angle (20**°**), and k is an arbitrary constant. As long as *T*_E _<< *T*_2_*, the exponential term containing *T*_2_* can be neglected. The constant k was determined for each individual VOI by dividing the baseline signal (before contrast arrival) during the rest scan by the remainder of the right-hand side of the equation evaluated for a *T*_10_ (baseline *T*_1_) of 1052 ms in myocardial tissue.^60^ For blood, a haematocrit-dependent *T*_10_ was used: *T*_10_ = 1000/(0.52 × HCT + 0.38).^61^ Then, for each myocardial segment and for blood the signal curve *S*(*t*) was converted to a *T*_1_ curve *T*_1_(*t*) by interpolating the *T*_1_ vs *S* curve. Gd-DOTA concentrations were subsequently calculated as:

$$ C_{\text{Gd}} \left( t \right) = \frac{1}{r}\left( {\frac{1}{{T_{1} \left( t \right)}} - \frac{1}{{T_{10} }}} \right) $$

where *r* is the relaxivity of Gd-DOTAREM: 2.8 s^−1^mM^−1^ at 3 T.^62^ Gd-DOTA concentrations during stress were corrected for Gd-DOTA remaining after the rest scan by subtracting the Gd-DOTA concentration prior to contrast arrival.

### Tracer Kinetic Model

Both PET and DCE-MRI data were analysed using the standard single-tissue compartment model (1TCM) with fitted blood volume:

$$ C_{T} \left( t \right) = \left( {1 - V_{\text{A}} } \right)K_{1} C_{\text{A}} \left( t \right) \otimes e^{{ - k_{2} t}} + V_{\text{A}} C_{\text{A}} \left( t \right) $$

For PET, the arterial input curve *C*_A_(*t*) was equal to the whole blood curve because of the free diffusibility of ^15^O-water whereas for DCE-MRI, *C*_A_(*t*) was the Gd-DOTA concentration in plasma, obtained using each individual’s hematocrite. For DCE-MRI, an additional parameter was added to account for the delay between the left ventricle and the arrival of the bolus in each myocardial segment. For ^15^O-water, an extra term *V*_RV_*C*_RV_(*t*) to account for spill-over from the right ventricular cavity was added, and *V*_A_ is rather a spill-over term than a blood volume term, which is why (1 − *V*_A_) was omitted in the first term of the equation. For both tracers, MBF was assumed equal to *K*_1_, and can then be interpreted as the transmural blood flow. Two different approaches to account for the limited extraction of Gd-DOTA were implemented. Firstly, a relationship between Gd-DOTA *K*_1_ values and ^15^O-water MBF values was obtained by fitting the Renkin-Crone model to the measured data:

$$ K_{1} = {\text{MBF}}\left( {1 - e^{{ - {\text{PS/MBF}}}} } \right) $$

where PS is the permeability surface area product and *K*_1_ values were converted to Gd DOTA MBF values by interpolation of this function. Secondly, the single-tissue compartment model was also implemented using permeability-surface area product PS as a fourth parameter (1TCM-PS) which allows for direct estimation of MBF:

$$ C_{T} \left( t \right) = \left( {1 - V_{\text{A}} } \right)MBF\left( {1 - e^{{ - {\text{PS/MBF}}}} } \right) \times C_{\text{A}} \left( t \right) \otimes e^{{ - k_{2} t}} + V_{\text{A}} C_{\text{A}} \left( t \right) $$

Since PS and MBF cannot be determined independently using a single fit, PS was determined using a coupled fit of rest and stress whole-myocardium Gd-DOTA concentration curves, assuming identical PS values during rest and stress scans, and then used as a fixed parameter for each individual segment.

For ^15^O-water, the model was fitted to the full 6 min of data, whereas for Gd-DOTA, data between the arrival of the bolus in the left-ventricular cavity and either the peak of the second passage of the bolus or the time at which the patient started breathing was used.

## Abbreviations

CAD: Coronary artery disease
CMR: Cardiac magnetic resonance
DCE: Dynamic contrast-enhanced
Gd: Gadolinium
MBF: Myocardial blood flow
MFR: Myocardial flow reserve
MR: Magnetic resonance
MRI: Magnetic resonance imaging
PS: Permeability surface
1TCM: One-tissue compartment model
