# Supplementary material for: Evaluation of quantitative CMR perfusion imaging by comparison with simultaneous 15O-water-PET
Source: J Nucl Cardiol. 2019 Jul 16;28(4):1252–66. doi: 10.1007/s12350-019-01810-z (PMC8421320; doi:10.1007/s12350-019-01810-z)
Supplement: Supplementary file 1 — Supplementary material 1 (PPTX 4481 kb) [file 12350_2019_1810_MOESM1_ESM.pptx]

## Slide 1
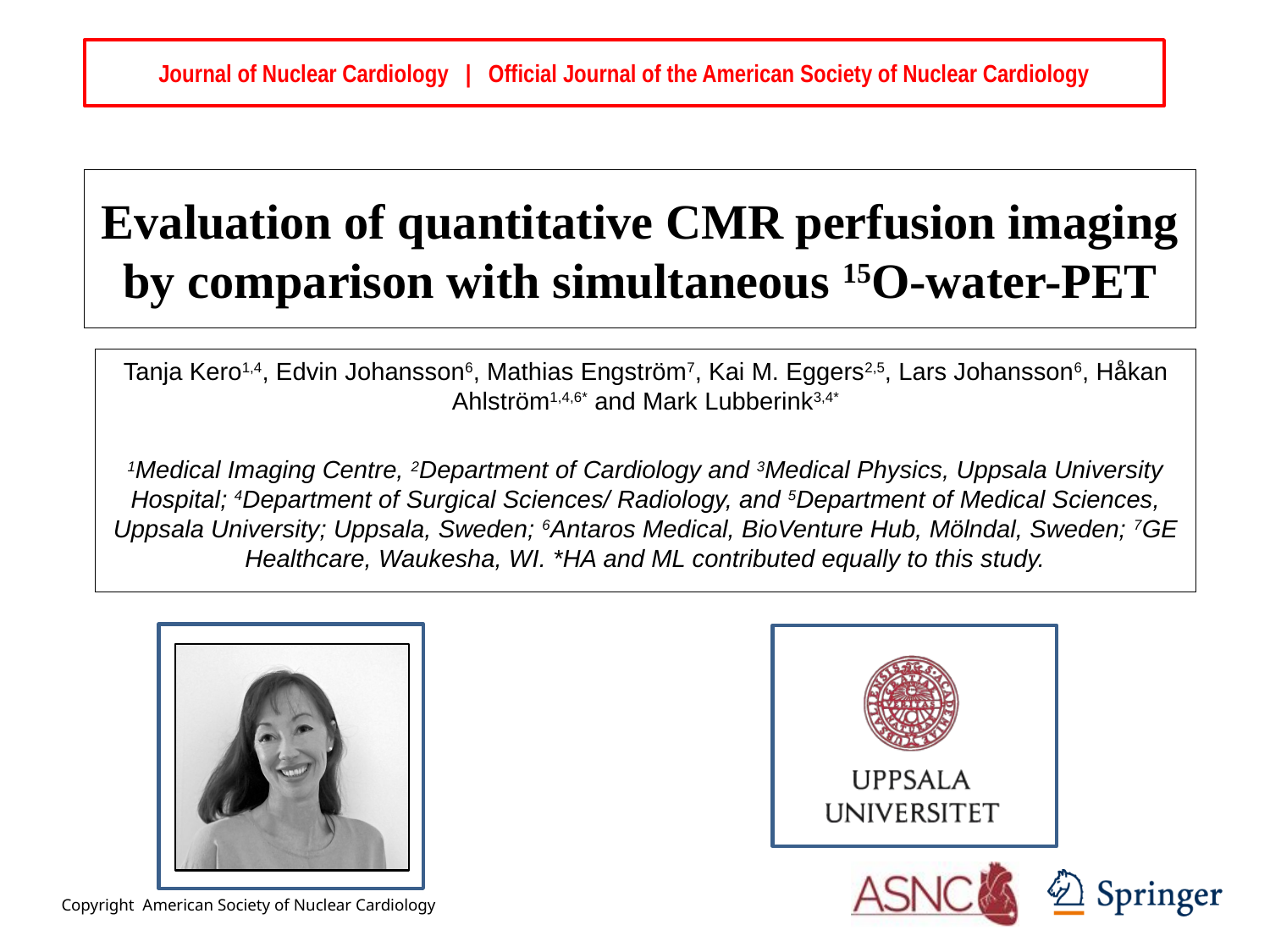

Journal of Nuclear Cardiology | Official Journal of the American Society of Nuclear Cardiology
# Evaluation of quantitative CMR perfusion imaging by comparison with simultaneous 15O-water-PET
Tanja Kero1,4, Edvin Johansson6, Mathias Engström7, Kai M. Eggers2,5, Lars Johansson6, Håkan Ahlström1,4,6* and Mark Lubberink3,4*
1Medical Imaging Centre, 2Department of Cardiology and 3Medical Physics, Uppsala University Hospital; 4Department of Surgical Sciences/ Radiology, and 5Department of Medical Sciences, Uppsala University; Uppsala, Sweden; 6Antaros Medical, BioVenture Hub, Mölndal, Sweden; 7GE Healthcare, Waukesha, WI. *HA and ML contributed equally to this study.
Copyright American Society of Nuclear Cardiology

## Slide 2
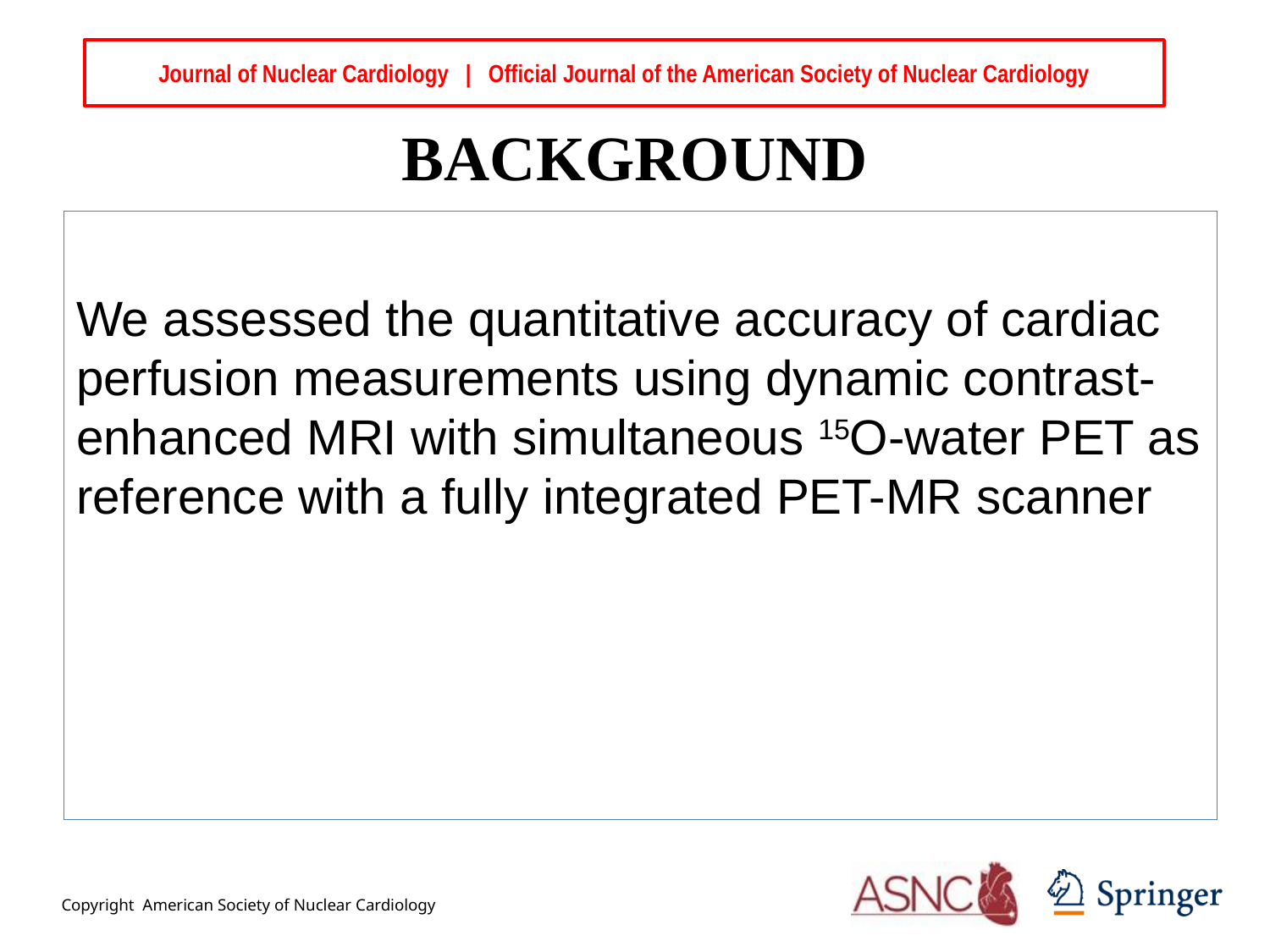

Journal of Nuclear Cardiology | Official Journal of the American Society of Nuclear Cardiology
# BACKGROUND
We assessed the quantitative accuracy of cardiac perfusion measurements using dynamic contrast-enhanced MRI with simultaneous 15O-water PET as reference with a fully integrated PET-MR scanner
Copyright American Society of Nuclear Cardiology

## Slide 3
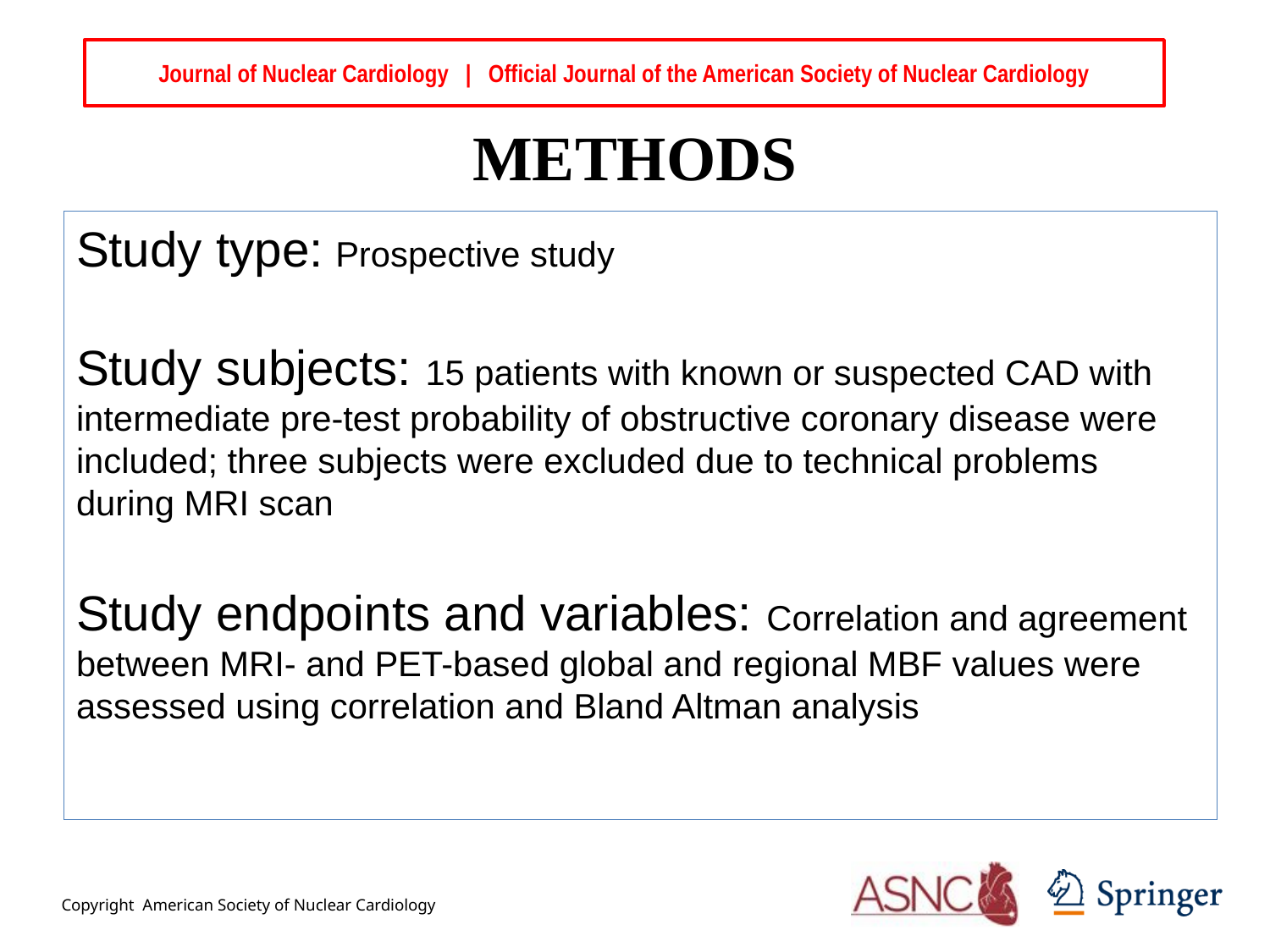

Journal of Nuclear Cardiology | Official Journal of the American Society of Nuclear Cardiology
# METHODS
Study type: Prospective study
Study subjects: 15 patients with known or suspected CAD with intermediate pre-test probability of obstructive coronary disease were included; three subjects were excluded due to technical problems during MRI scan
Study endpoints and variables: Correlation and agreement between MRI- and PET-based global and regional MBF values were assessed using correlation and Bland Altman analysis
Copyright American Society of Nuclear Cardiology

## Slide 4
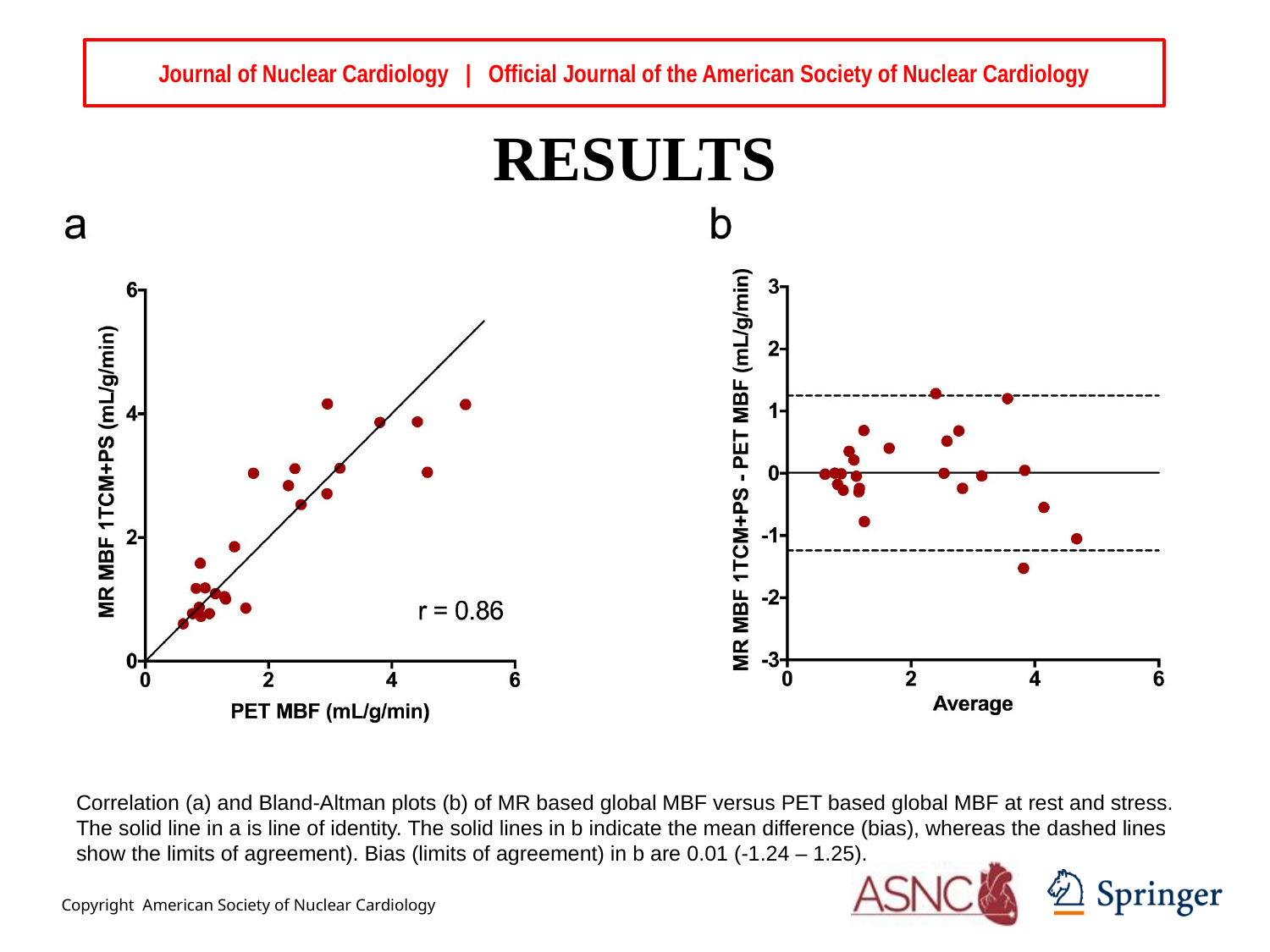

Journal of Nuclear Cardiology | Official Journal of the American Society of Nuclear Cardiology
# RESULTS
Correlation (a) and Bland-Altman plots (b) of MR based global MBF versus PET based global MBF at rest and stress. The solid line in a is line of identity. The solid lines in b indicate the mean difference (bias), whereas the dashed lines show the limits of agreement). Bias (limits of agreement) in b are 0.01 (-1.24 – 1.25).
Copyright American Society of Nuclear Cardiology

## Slide 5
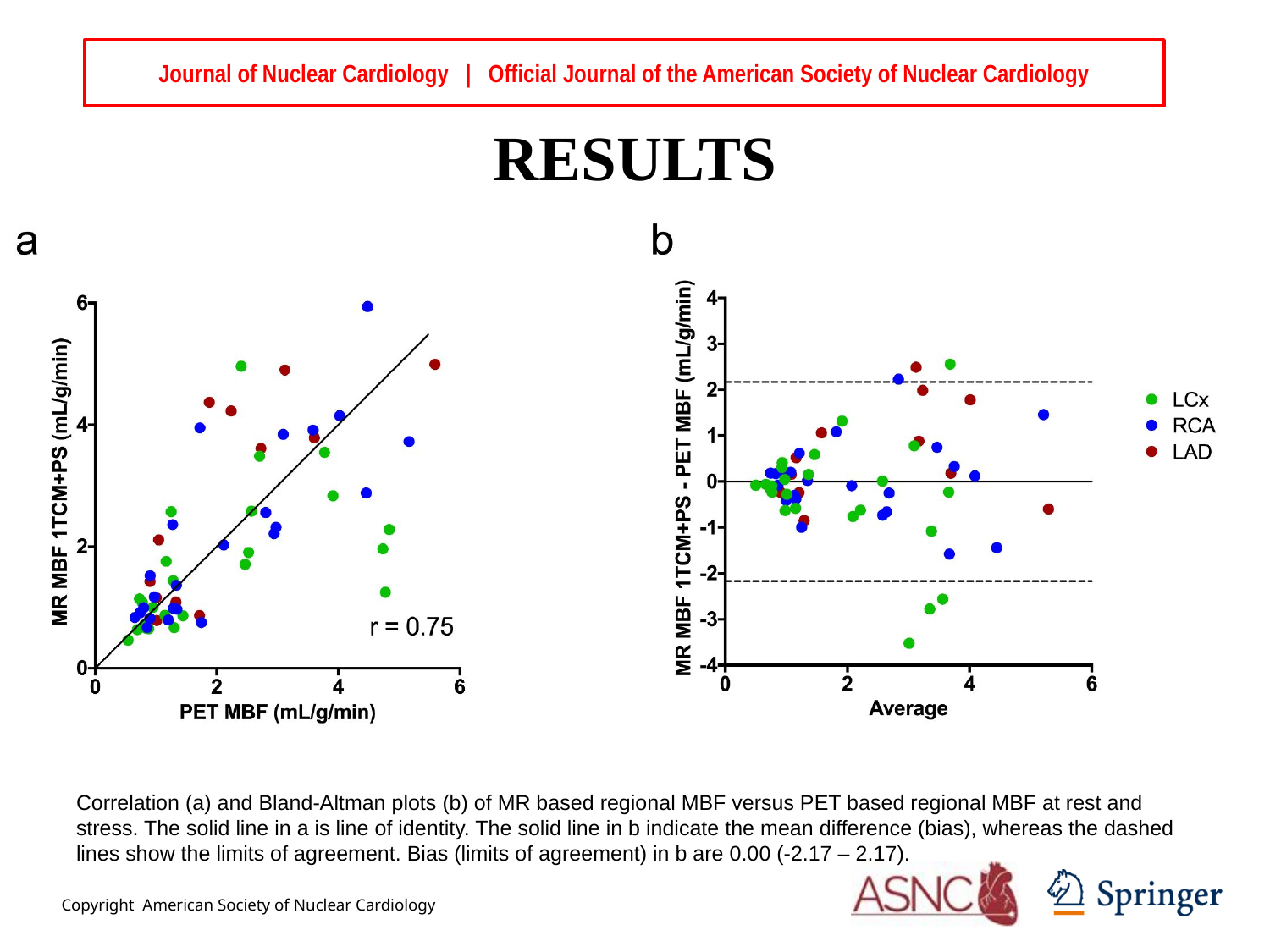

Journal of Nuclear Cardiology | Official Journal of the American Society of Nuclear Cardiology
# RESULTS
Correlation (a) and Bland-Altman plots (b) of MR based regional MBF versus PET based regional MBF at rest and stress. The solid line in a is line of identity. The solid line in b indicate the mean difference (bias), whereas the dashed lines show the limits of agreement. Bias (limits of agreement) in b are 0.00 (-2.17 – 2.17).
Copyright American Society of Nuclear Cardiology

## Slide 6
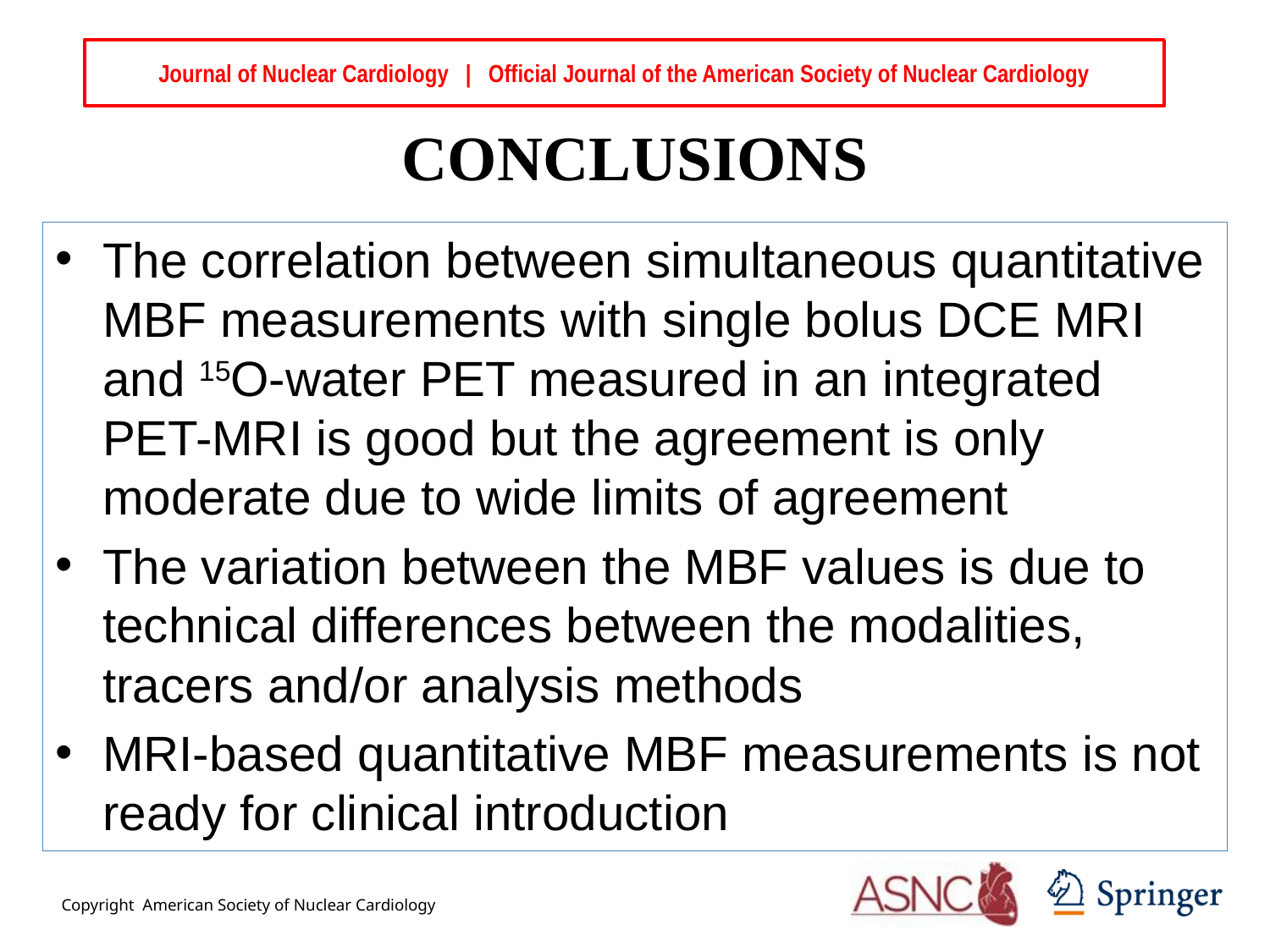

Journal of Nuclear Cardiology | Official Journal of the American Society of Nuclear Cardiology
# CONCLUSIONS
The correlation between simultaneous quantitative MBF measurements with single bolus DCE MRI and 15O-water PET measured in an integrated PET-MRI is good but the agreement is only moderate due to wide limits of agreement
The variation between the MBF values is due to technical differences between the modalities, tracers and/or analysis methods
MRI-based quantitative MBF measurements is not ready for clinical introduction
Copyright American Society of Nuclear Cardiology
